# Quantification of multiple steroid hormones in serum and human breast cancer tissue by liquid chromatography-tandem mass spectrometry analysis

**DOI:** 10.3389/fonc.2024.1383104

**Published:** 2024-05-28

**Authors:** Feng Wang, Eline Eikeland, Randi J. Reidunsdatter, Lars Hagen, Monica J. Engstrøm, Jürgen Geisler, Mikko Haanpää, Esa Hämäläinen, Guro F. Giskeødegård, Tone F. Bathen

**Affiliations:** ^1^ Department of Circulation and Medical Imaging, Norwegian University of Science and Technology, Trondheim, Norway; ^2^ Department of Breast and Endocrine of Surgery, St. Olavs Hospital, Trondheim University Hospital, Trondheim, Norway; ^3^ Department of Clinical and Molecular Medicine, Norwegian University of Science and Technology, Trondheim, Norway; ^4^ Clinic of Laboratory Medicine, St. Olavs Hospital, Trondheim, Norway; ^5^ PROMEC Core Facility for Proteomics and Modomics, Norwegian University of Science and Technology, and the Central Norway Regional Health Authority Norway, Trondheim, Norway; ^6^ Department of Oncology, Akershus University Hospital, Lørenskog, Norway & Institute of Clinical Medicine, Faculty of Medicine, University of Oslo, Oslo, Norway; ^7^ HUSLAB, Helsinki University Hospital, Helsinki, Finland; ^8^ Department of Clinical Chemistry, University of Eastern Finland, Kuopio, Finland; ^9^ Department of Public Health and Nursing, Norwegian University of Science and Technology, Trondheim, Norway; ^10^ Department of Radiology and Nuclear Medicine, St. Olavs Hospital, Trondheim University Hospital, Trondheim, Norway

**Keywords:** breast cancer, steroid hormone, LC-MS/MS, quantification, breast cancer tissue

## Abstract

**Introduction:**

Systemic and local steroid hormone levels may function as novel prognostic and predictive biomarkers in breast cancer patients. We aimed at developing a novel liquid chromatography-tandem mass spectrometry (LC-MS/MS) method for the simultaneous measurement of multiple, biologically pivotal steroid hormones in human serum and breast cancer tissue.

**Methods:**

The quantitative method consisted of liquid-liquid extraction, Sephadex LH-20 chromatography for tissue extracts, and analysis of steroid hormones by liquid-chromatography-tandem mass spectrometry. We analyzed serum and tissue steroid hormone levels in 16 and 40 breast cancer patients, respectively, and assessed their correlations with clinical parameters.

**Results:**

The method included quantification of nine steroid hormones in serum [including cortisol, cortisone, corticosterone, estrone (E1), 17β-estradiol (E2), 17α-hydroxyprogesterone, androstenedione (A4), testosterone and progesterone) and six (including cortisone, corticosterone, E1, E2, A4, and testosterone) in cancer tissue. The lower limits of quantification were between 0.003–10 ng/ml for serum (250 µl) and 0.038–125 pg/mg for tissue (20 mg), respectively. Accuracy was between 98%-126%, intra-assay coefficient of variations (CV) was below 15%, and inter-assay CV were below 11%. The analytical recoveries for tissue were between 76%-110%. Tissue levels of E1 were positively correlated with tissue E2 levels (p<0.001), and with serum levels of E1, E2 and A4 (p<0.01). Tissue E2 levels were positively associated with serum E1 levels (p=0.02), but not with serum E2 levels (p=0.12). The levels of tissue E2 and ratios of E1 to A4 levels (an index for aromatase activity) were significantly higher in patients with larger tumors (p=0.03 and p=0.02, respectively).

**Conclusions:**

The method was convenient and suitable for a specific and accurate profiling of clinically important steroid hormones in serum. However, the sensitivity of the profile method in steroid analysis in tissue samples is limited, but it can be used for the analysis of steroids in breast cancer tissues if the size of the sample or its steroid content is sufficient.

## 1 Introduction

The majority of human breast cancers are hormone-dependent and express estrogen and/or progesterone receptors (ER and/or PR) (1). Elevated estrogen blood levels as well as high localized estrogen tissue levels in the breast have been associated with an increased breast cancer risk (2, 3). However, estrogen and androgen levels are not routinely measured in breast cancer patients, although systemic and local steroid hormone levels might function as novel biomarkers for a more precise disease diagnosis. Sensitive and fast methods for measuring biologically pivotal steroid hormones are needed during endocrine treatment with aromatase inhibitors or gonadotropin releasing hormone-analogues to evaluate the individual effect on steroid metabolism as well as compliance.

Steroid hormones and their metabolites exist in biofluid and biological tissues at very low levels, with a large dynamic range from picomolar to micromolar concentrations. Liquid chromatography tandem mass spectrometry (LC-MS/MS) is considered as the gold standard for quantifying endogenous steroid hormones in serum, and also the preferred analytical approach for tissue samples (4, 5). However, both LC-MS/MS and gas chromatography tandem mass spectrometry (GC-MS/MS) have inadequate sensitivity for quantifying steroid hormones in a limited amount of sample. In particular, due to the complexity of tissue, it is more difficult to quantify steroid hormones in tissue than in serum (6, 7). Sample preparation and concentrating steroid hormones prior to mass spectrometry analysis are the key steps for validating a quantitative method. Currently, only a limited number of mass spectrometry (MS)-based methods for simultaneously analyzing steroid hormones and their metabolites in breast cancer tissue or breast adipose tissue samples have been reported (**Supplementary Table 1**) (3, 5–16). Most of them either acquired a large amount of tissue samples, included a derivatization step before MS analysis, or analyzed few steroid hormones. In addition, due to different procedures for sample preparation and analytical approaches, it is not easy to directly compare the results among different studies and to validate the findings by use of a separate patient cohort (4).

In this study, we have developed a new LC-MS/MS method for simultaneously measuring steroid hormones in both serum and breast cancer tissue. We further applied this method to quantify steroid hormones in serum and cancer tissue from a pilot cohort of breast cancer patients, and assessed their association with clinical parameters.

## 2 Materials and methods

### 2.1 Patient cohort and sample material

The pilot cohort included 40 patients with ER-positive breast cancer from the Regional Breast Cancer Biobank in Mid-Norway. The cancer tissue were collected during tumor excision and immediately stored in liquid-nitrogen until analysis. All the patients had invasive breast cancer and underwent surgical removal of the tumor at St. Olav Hospital in Trondheim, Norway, between 1999–2014. The guidelines from the Norwegian Breast Cancer group for subsequent treatment regime at that time were followed (17). In addition, serum samples were available from 16 out of these 40 patients, who also participated in an observational study (18). The serum samples were taken after breast cancer surgery, and before the start of radiation treatment, and stored in -80°C until analysis. The study was granted approval by the Regional Committee for Medical and Health Sciences Research Ethics (REK, Midt-Norge, ID 2019/520 and ID 125405, and REK 2019/13760). All patients signed informed consents.

### 2.2 Steroid hormones, chemicals, calibrators and quality control samples

Steroid hormones [E2, E1, 17α-hydroxyprogesterone (17OHP), cortisol (F), cortisone (E), corticosterone (CORT), A4, T, and progesterone (P4)] and deuterated steroid hormones [17β-estradiol-d4 (d4-E2), estrone-d4 (d4-E1), androstenedione-d7 (d7-A4), testosterone-d3 (d3-T), and progesterone-d9 (d9-P4)] were bought from Chiron AS (Oslo, Norway). Cortisone-d8 (d8-E) and corticosterone-d8 (d8-CORT) were obtained from Toronto Research Chemicals (Toronto, Canada), while Cortisol-d4 (d4-F) and 17α-hydroxyprogesterone-d8 (d8–17OHP) were purchased from Sigma-Aldrich (St. Louis, USA). Stock solutions of steroid hormones (1 mg/ml or 100 μg/ml) were dissolved in methanol (MeOH) and stored at -20°C.

Ammonium fluoride (NH_4_F), acetonitrile, ethyl acetate, toluene, and MeOH (HLPC grade) was bought from Sigma-Aldrich (St. Louis, USA), while MeOH and water (Optima LC/MS) were from Fisher Scientific (Oslo, Norway). *n*-hexane (HX) and Methyl tert-butyl ether (MTBE) were purchased from Merck (Darmstadt, Germany).

The concentrations of eight-point calibration curves, three quality control (QC) samples, as well as samples of lower limits of quantification (LLOQs) are listed in **Supplementary Table 2**. Steroid hormone working solutions with 100 times higher concentrations than those in calibrators and quality control samples, were prepared in MeOH. After that, one percent (1:99, v: v) of each working solution was spiked into steroid-free serum (BBI Solutions, UK) to make both calibrators and quality control samples. After mixing for 10 mins, the samples were divided into 300 µl aliquots and stored at -20°C. The internal standard mixture consisted of nine deuterated steroid hormones prepared in MeOH/water (1:1, v:v) and stored at -20°C.

### 2.3 Sample preparation

**Figure 1:**
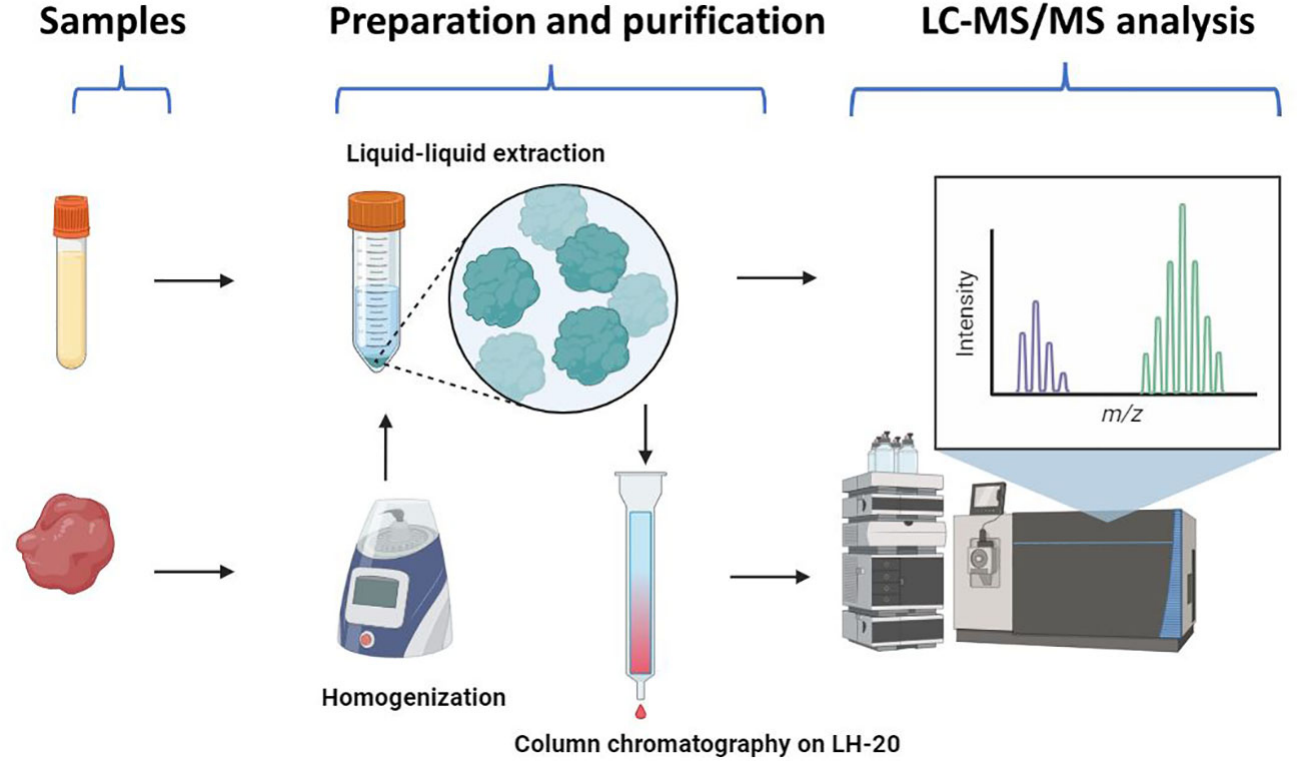
The workflow of the LC-MS/MS method for steroid profiling. The serum method consisted of liquid-liquid extraction and LC-MS/MS analysis. The tissue method included homogenization, liquid-liquid extraction, and purification by column chromatography on Sephadex LH-20, prior to LC-MS/MS analysis. Created with Biorender.com.

**Figure 1** shows the outlet of the quantification method. The tissue method was slightly modified from the serum method, in which one purification step with column chromatography on Sephadex LH-20 (Merck, Darmstadt, Germany) was applied to remove the lipid impurities from tissue extracts. In addition to serum/tissue samples. each assay contained two blank samples (steroid-free serum), eight calibrators, as well as six quality control samples.

#### 2.3.1 Serum

We used a liquid-liquid extraction with HX/MTBE for steroids in serum samples. After adding 20 µl of the internal standard mixture (concentrations seen in **Supplementary Table 2**), 250 µl of serum samples were mixed with 1 ml of HX/MTBE (3:1 v/v) for 10 mins, and then incubated at room temperature for 30 mins. One blank sample was proceeded without the internal standard mixture. The samples were centrifugated at 3000 rpm for 10 mins and the organic phase in each sample was collected. The extraction was repeated once and both extracts were mixed together. After evaporation of the organic solvent, the sample was dissolved in 50 µl of MeOH/water (1:1, v/v).

#### 2.3.2 Tissue

Breast cancer tissue samples (approximately 20–35 mg) were cut on a working station, cooled by liquid nitrogen and then transferred to Precellys^®^ 2 ml Tissue Homogenizing Mixed Beads Kit (Bertin instrument) containing 250 µl of water. After adding 20 µl internal standard mixture, samples were homogenized in Precellys 24 homogenizer (Bertin instruments, 6000 rpm, 6 intervals). The homogenized tissue samples, as well as two blank samples, eight calibrators, and six quality control samples, were extracted by liquid-liquid extraction as described above for serum samples. To remove the lipid impurities, Sephadex LH-20 column chromatography that is one type of partition chromatography and can be used to separate steroid hormones in terms of their polarity, was performed as follows. The samples were evaporated to dryness, re-dissolved in 300 µl HX, and applied to the LH-20 columns (0.5 cm× 6 cm). Lipid impurities were washed out with 4 ml of HX and discarded. Some of steroid hormones, which were not well dissolved in HX and thus might remain in the sample tubes, were washed with 300 µl MeOH and transferred to the LH-20 columns for two times. Consequently, five ml of MeOH was used to elute the steroid hormones from the LH-20 columns. After evaporating to dryness, the samples were dissolved in 50 µl of MeOH/water (1:1, v/v).

### 2.4 LC-MS/MS analysis

LC-MS/MS experiments were carried out by the mass spectrometer by a Turbo V™ Ion Source (API 5500 triple quadrupole mass spectrometer, AB Sciex, Concord, CA). Peripherals consisted of a Prominence Series HPLC system with a LC-20AD binary pump (Shimadzu, Kyoto, Japan). Separation was carried out by a tandem column system where a Discovery HS F5 Superguard column (20 mm × 2.1 mm, 3 µm, Merck Life Science AS, Norway) and a Discovery HS F5 HPLC column (100 mm × 2.1 mm, 3µm, Merck Life Science AS, Norway) were coupled with a SunFire C18 column (50 × 2.1 mm, 3.5 μm, Waters, Milford, MA). The use of the second HPLC column enhances the total chromatographic separation, resulting in improved peak definition in mass spectrometry chromatograms and a significant reduction in background noise during MS detection. The mobile phase gradient and multiple transition monitoring (seen in **Supplementary Table 3**) were described by Häkkinen et al, with some modifications (15). The mobile phase was a linear gradient consisting of 0.1 mM NH_4_F in water (mobile phase A) and 0.1 mM NH_4_F methanol: water (99:1, vol: vol) (mobile phase B), at a flow rate of 200 μl/min. The gradient was as follows: 0 min, 40% mobile phase B; 0–3.5 min, linearly increased to 68% mobile phase B; 3.5–9.5 min, from 68% to 71% mobile phase B; 9.5–13.5 min, from 71% to 80% mobile phase B; 13.5–14.5 min, from 80% to 100% mobile phase B; 14.5–19.1 min, 100% mobile phase B; and 19.1–21 min, linearly decreased to 40% mobile phase B. The injection volumes were 10 µl for the negative mode and 5 µl for the positive mode, respectively. The source optimization was prioritized to the analysis of E2 in the negative mode and A4 in the positive mode: curtain gas: 20 and 30, collision gas: 12 and 9, ion spray voltage: -4000 and 5250, ion source gas 1: 60 and 60, and ion source gas 2: 40 and 50. The temperature was 550 ˚C. The limits of detection (LOD) were defined as signal to noise ratio (S/N) ≥ 3. Data were acquired and processed with Analyst Software (version 1.7.2, AB Sciex). The criteria for accepting a run was based on “ICH guideline M10 on bioanalytical method validation” (19).

### 2.5 Analysis of endogenous steroid hormone concentrations in serum and breast cancer tissue in patients with breast cancer

The serum concentrations of steroid hormones in 16 patients and cancer tissue samples from 40 patients were measured by the described methods. The tissue steroid hormone concentrations were adjusted by the tissue weight: steroid hormone concentration in tissue (pg/mg) = concentration measured by LC-MS/MS (ng/ml) × 0.25 (ml) × 1000 (pg/ng)/weight (mg).

### 2.6 Statistical analysis

Statistical analysis performed in Matlab R2021b. Data were expressed as mean ± SD, or median (range). Steroid hormone concentrations whose values were below LLOQ, were replaced with imputed data obtained by a compositional approach using the R package zComp (20). The endogenous concentrations of serum and tissue steroid hormones were not normally distributed, assessed by qq-plots. Association of tissue and serum steroid hormone concentrations, and of tissue steroid hormone levels and clinical characteristics, such as grade, nodal spread status, and BMI, were evaluated by Spearman’s rank correlation. Between-group (normal weight vs. overweight, and breast cancer recurrence vs. non-recurrence) differences were analyzed with the independent-samples Mann-Whitney U test.

## 3 Results

### 3.1 Assay characteristics

#### 3.1.1 The serum method

**Figure 2:**
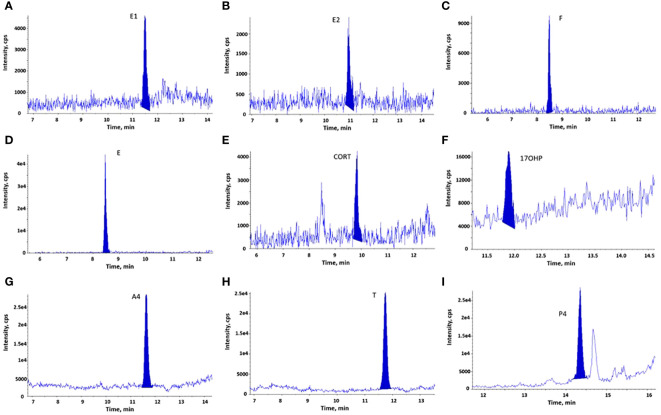
LC-MS/MS chromatograms of steroid hormones measured in serum samples (LLOQs or calibrators). **(A)**. E1; **(B)**. E2; **(C)**. F (calibrator 1); **(D)**. E (calibrator 1); **(E)**. CORT (calibrator 3); **(F)**. 17OHP (calibrator 2); **(G)**. A4; **(H)**. T; **(I)**. P4. Both calibrators and quality control samples (including LLOQs) were prepared by spiking steroid hormones into steroid-free serum, as described in section 2.2. LLOQs represent samples for lower limits of quantification.

The serum method can quantify nine steroid hormones (F, E, CORT, E1, E2, 17OHP, T, P4, and A4) in serum, which was optimized by prioritizing E2 and E1. The chromatographic separation of the nine steroid hormones were obtained (**Figure 2**). In addition, both 17OHP and T could be chromatographically separated from their epimers (17β-hydroxyprogesterone and epitestosterone, respectively, **Supplementary Figure 1**).

The LC-MS/MS method was linear at concentrations of 0.005–0.4 ng/ml for E2 and E1, 2–50 ng/ml for E, 1–60 ng/ml for CORT (calibrators 3–8), and 0.05–5 ng/ml for 17OHP (calibrators 2–8). It was quadratic regression at concentrations of 10–500 ng/ml for F, 0.05–5 ng/ml for A4, 0.05–2.5 ng/ml for T, and 0.02–5 ng/ml for P4. The correlation coefficient was greater than 0.997 for all the analytes with a weighting of 1/x.

The sensitivity, accuracy and precision of the serum method were determined in three validation assays, while each assay contained three levels of QC samples (six replicate samples for each level), as well as LLOQs (**Supplementary Table 4**). Both intra-assay and inter-assay coefficient variation (CV) were calculated by dividing the mean by the standard deviation (SD) and then multiplying by 100%. The intra-assay CV was calculated from six replicate samples within each assay and the mean ± SD of the three intra-assay CVs were presented. The inter-assay CV was calculated from the mean concentrations derived from six replicate samples in each of the three assays. LLOQ for steroid hormones were determined based on the concentrations measured in six replicate samples (LLOQs) in each of the three validation assays, with a CV smaller than 20%. It was acceptable for five steroid hormones (E2, E1, A4, T, and P4), with accuracy ranging of 86%-115% (except for E2 in one assay, 122%). The other four steroid hormones were either not detectable in LLOQs (CORT and 17OHP) or their accuracies were low (F and E). Hence, the concentrations of F and E in calibrator 1, of 17OHP in calibrator 2, and of CORT in calibrator 3, were considered as their respective LLOQ. In summary, LLOQ for E2, E1, F, E, CORT, 17OHP, A4, T and P4 was 0.005, 0.003, 10, 2, 1, 0.05, 0.03, 0.03, and 0.02 ng/ml, respectively. The mean accuracies were 98.4%-113.4%, except for T in QC1 (125.7%). All inter-assay CVs were below 10.6%. The means of intra-assay CVs varied between 2.3% to 11.7% for eight steroid hormones, while it was higher (6.9%-15.4%) for F.

#### 3.1.2 The tissue method

**Figure 3:**
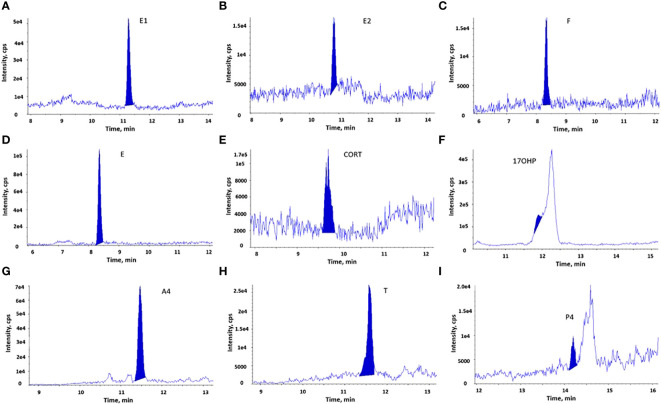
LC-MS/MS chromatograms of steroid hormones measured in breast cancer tissue extracts. The chromatographic separation of 9 steroid hormones is observed in the breast cancer tissue extracts. **(A)**. E1; **(B)**. E2; **(C)**. F; **(D)**. E; **(E)**. CORT; **(F)**. 17OHP, an interfering peak with the similar retention time hindered the detection of 17OHP; **(G)**. A4; **(H)**. T; **(I)**. P4.

Column chromatography on LH-20 was able to remove lipids from the tissue extracts. In total, our method can accurately quantify six steroid hormones (E, CORT, E1, E2, T, and A4) in tissue. Like serum extracts, the steroid hormones extracted from tissue were identified by their chromatograms (**Figure 3**). However, we observed an interfering peak with similar retention time for 17OHP in tissue extracts, which made it impossible to quantify 17OHP. The LLOQ for the other eight steroid hormones in the tissue method remained the same as those in the serum method. In addition, regression correlations (r > 0.998) of calibrator curves and accuracies of six QC samples for these eight steroid hormones were within acceptable ranges.

The analytical recovery of the tissue method was determined by spiking steroid hormones into homogenized breast cancer tissue, which is showed in **Supplementary Table 5**. Six steroid hormones had acceptable recoveries (76%−110%), while the mean recovery was low (64.9%) for P4 and high for F (145.5%). The recoveries of F and E were not affected by the concentrations of internal standards or volumes injected to the LC-MS/MS analysis. In addition, the reproducibility of the tissue method was good for these steroid hormones. The means of inter-assay CV in three assays (n=3 in each assay) varied between 2.5% and 8.6%, and intra-assay CV were between 3.9% and 17.0%.

### 3.2 Concentrations of steroid hormones in serum and cancer tissue in breast cancer patients

The clinical characteristics of 40 patients with ER-positive breast cancer is shown in **Table 1**. Since there was only one premenopausal woman in the cohort, the menopausal status was not considered in the analysis. The concentrations of six steroid hormones in serum and breast cancer tissue are shown in **Table 2**. In breast cancer tissue, three steroid hormones (E1, E2, and A4) were quantifiable and the other three steroid hormones (E, CORT, and T) were not detectable. Tissue E2 and E1 levels were positively correlated (Spearman r=0.65, p<0.001), but none of them were correlated to BMI or ER (%) (p>0.05). Tissue E2 levels and the ratio of E1 to A4 concentrations were significantly higher in patients with a larger tumor size (2–5 cm, n=22) compared to those with a smaller tumor size (<2 cm, n=17) (median concentration, 0.41 vs. 0.14 pg/mg, p=0.03). However, these tissue steroid hormone levels did not significantly differ between patients with different grades, nodal spread status, BMI, or with and without breast cancer recurrence.

**Table 1:** The clinical characteristics in the patient cohort.

		The whole patient cohort	Normal weight group	Overweight and obese group
		(n=40)	(n=20, 20≤BMI<25)	(n=20, BMI≥25)
Age (mean ± SD), years		65.8 (± 9.0)	66.1 (± 9.6)	65.5 (± 8.5)
BMI (mean ± SD), kg/m^2^		27.0 (± 5.6)	22.9 (± 1.3)	31.1 (± 5.3)
Grade (n)	1	4	3	1
	2	26	10	16
	3	10	7	3
Tumor size (n)	<2 cm	17	11	6
	2–5 cm	22	9	13
	>5 cm	1	0	1
Nodal spread (n)	0	22	14	8
	≥1	18	6	12
ER (n)	Positive*	40	20	20
ER (mean± SD)*	%	79.1 (± 21.5)	78.8 (± 22.0)	79.4 (± 21.7)
PR (n)	Negative	6	2	4
	Positive*	34	18	16
Recurrence (n)	No	31	16	15
	Yes	9	4	5

SD, standard deviation. BMI, body mass index. Six patients (three per BMI group) had no values for ER percent. *, cut-off values for the ER- and the PR-positive were >10%.

**Table 2:** Tissue and serum concentrations of steroid hormones.

Steroid	Tissue (median, range, pg/mg)	Serum (median, range, ng/ml)
	n=39*	n=16
E1	0.093 (0.026–1.607)	0.019 (0.002–0.115)
E2	0.208 (0.001–1.150)	0.003 (0.001–0.041)
E	N.D.**	15.8 (9.1–30.0)
CORT	N.D.**	N.D.**
A4	0.696 (0.003–8.080)	0.453 (0.148–1.560)
T	N.D.**	0.19 (0.07–1.15)

The concentrations of E2, E1, A4 in breast cancer tissue were below the LLOQ in 35%, 10%, and 20% of 40 patients, respectively. The levels of E2 and E1 in serum were below the LLOQ in 62.5% and 6.3% of 16 patients, respectively. For those below LOQs, data were imputed by a compositional approach using the R package zComp. *, One patient was excluded because all the three tissue steroids were below LOQs. **, not detectable.

**Figure 4:**
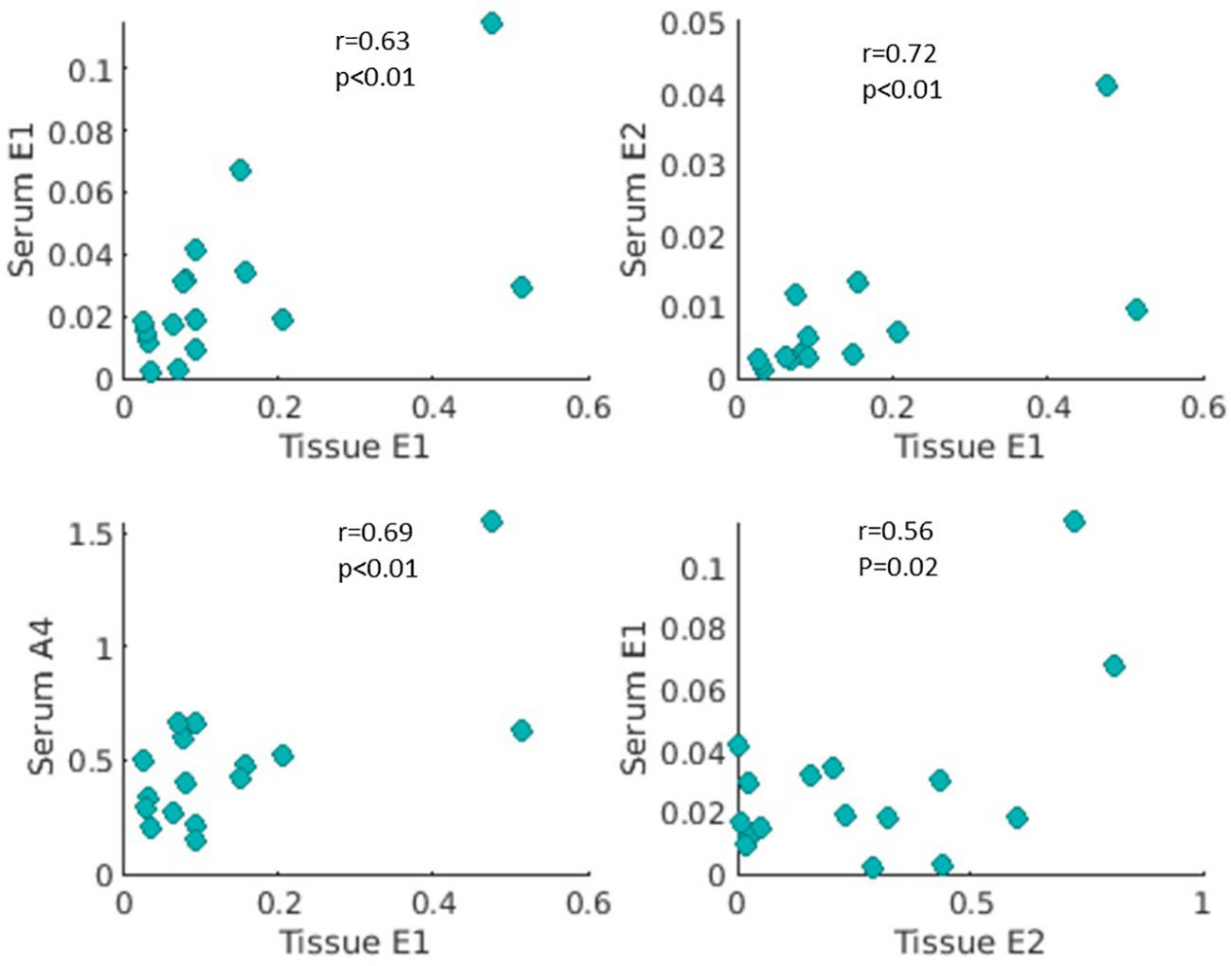
Correlations of estrone (E1), 17β-estradiol (E2) and androstenedione (A4) in serum and breast cancer tissue extracts. Levels of E2, E1, and A4 were measured in both serum and breast cancer tissue in 16 patients with ER-positive breast cancer. Tissue E1 concentration (pg/mg) was positively correlated with serum concentrations (ng/ml) of E1, E2, and A4 (r=0.63, 0.72, 0.69, and p<0.01, respectively). Tissue E2 level (pg/mg) was positively associated with serum E1 level (ng/ml) (r=0.52, p=0.02).

In 16 patients who had both serum and tissue steroid hormone levels available, the tissue levels of E1 were positively correlated with serum E1, E2 and A4 levels (Spearman correlation, *r*= 0.63, *r*= 0.72, *r* =0,69, respectively, *p*<0.01 for all three, **Figure 4**). The tissue levels of E2 were positively correlated with serum E1 levels (*r*=0.56, *p*= 0.02, **Figure 4**), but not with serum E2 and A4 levels (*p*= 0.12 and *p*= 0.10, respectively).

## 4 Discussion

In this study, we validated a novel LC-MS/MS-based-method for simultaneously measuring nine steroid hormones in serum and six steroid hormones in human breast cancer tissue. The advantages of our method include a broad range of steroid hormones (two estrogens, two androgens, and two glucocorticoids) covered, no derivatization step needed, and fast analysis (21). Similar to adipose tissue, breast cancer tissue may contain high concentrations of lipids with similar physical and chemical characteristics to steroid hormones, which cause ion suppression or interference in the steroid signals in mass spectrometry analysis (7, 21). We found that column chromatography on Sephadex LH-20, after the liquid-liquid extraction with the mixture of HX and MTBE, was the best choice to clean-up the tissue extracts and remove these interfering substances from our tissue samples. We obtained good accuracies for nine and six steroid hormones in serum and tissue extracts, respectively. However, an unknown interfering compounds hindered the analysis of 17OHP in tissue extracts. Also the poor accuracies for F and P4 in the tissue extracts could not be totally explained by matrix effects (22, 23). Further studies are needed to include these three steroid hormones in the method.

The range of tissue E2, E1, and A4 concentrations in our cohort was consistent with previous data (3, 24). We found that tissue concentration of E2 and the ratio of E1 to A4 were significantly higher in patients with larger tumors, which were in agreement with previous studies (3, 8). These support the theory that high levels of estrogen in the breast may contribute to the risk of ER-positive breast tumors and its growth (25). Although tissue and serum samples were not collected at the same timepoint in our cohort, tissue levels of E1 were positively correlated with serum E1, E2 and A4 levels. However, we did not find any significant correlation between cancer tissue steroid concentrations and BMI or breast cancer recurrence. In contrast, several previous studies have reported positive correlations between tissue steroid hormone concentrations and BMI (26, 27). Obesity was reportedly negatively associated with aromatase inhibitor efficiency in postmenopausal ER-positive breast cancer (28). Since breast cancer is a highly heterogeneous disease and tissue levels of endogenous steroid hormones are considerably variable during menstrual cycle and due to the menopause (29), our cohort might have been too small to achieve significant results.

In breast adipose tissue of patients with breast cancer, the median concentration of E was 2 or 4 times higher than those of E1 or E2 (30). To our best knowledges, concentrations of E and CORT in human breast cancer tissue have not been analyzed by MS-based methods previously. We did not find quantifiable E and CORT in our cohort. However, the transfer of very low percentage of corticosteroids to adjacent breast cancer tissue cannot be ruled out.

The sensitivity of our method is adequate for analysis of serum estrogens in pre-menopausal women, but not for all post-menopausal women (usually varying between 0.003 ng/ml to 0.011–0.016 ng/ml). To quantify very low concentrations of serum estrogens in breast cancer patients receiving aromatase inhibitor treatment, MS-methods that are more sensitive and more specific for estrogen analysis have been developed (31, 32). Our method is sensitive for analyzing serum androgens in both men and women, but it may not be sensitive enough for measuring tissue androgens when such a small amount of cancer tissue was used. This may partially explain our observation where tissue levels of E, CORT, and T in our cohort were not detectable.

In conclusion, our method was convenient and suitable for a specific and accurate profiling of clinically important steroids in serum. However, the analysis of some steroids (such as P4, T and F) in breast cancer tissue appeared to be challenging, likely due to the small size of our breast cancer samples. Analyzing steroids in tissue samples with our methods could, however, be feasible if the sample size and steroid content are sufficient. As expected, tissue levels of E1 were positively correlated with tissue levels of E2, and serum levels of E1, E2, and A4. We observed a trend that patients with large tumor diameter, had significantly higher concentrations of E2 in their breast cancer tissue.

## Data availability statement

The original contributions presented in the study are included in the article/**Supplementary Material**. Further inquiries can be directed to the corresponding authors.

## Ethics statement

The study was granted approval by the Regional Committee for Medical and Health Sciences Research Ethics (REK, Midt-Norge, ID 2019/520 and ID 125405, and REK 2019/13760). The studies were conducted in accordance with the local legislation and institutional requirements. The participants provided their written informed consent to participate in this study.

## Author contributions

FW: Conceptualization, Data curation, Funding acquisition, Investigation, Methodology, Project administration, Writing – original draft, Writing – review & editing. EE: Data curation, Writing – review & editing. RR: Writing – review & editing. LH: Methodology, Writing – review & editing. ME: Writing – review & editing. JG: Methodology, Writing – review & editing. MH: Methodology, Writing – review & editing. EH: Methodology, Writing – review & editing. GG: Investigation, Writing – review & editing. TB: Conceptualization, Writing – review & editing, Funding acquisition, Supervision.
