# Supplementary material for: Quantification of multiple steroid hormones in serum and human breast cancer tissue by liquid chromatography-tandem mass spectrometry analysis
Source: Front Oncol. 2024 May 28;14:1383104. doi: 10.3389/fonc.2024.1383104 (PMC11165045; doi:10.3389/fonc.2024.1383104)
Supplement: Supplementary file 1 [file Presentation_1.pptx]

## Slide 1
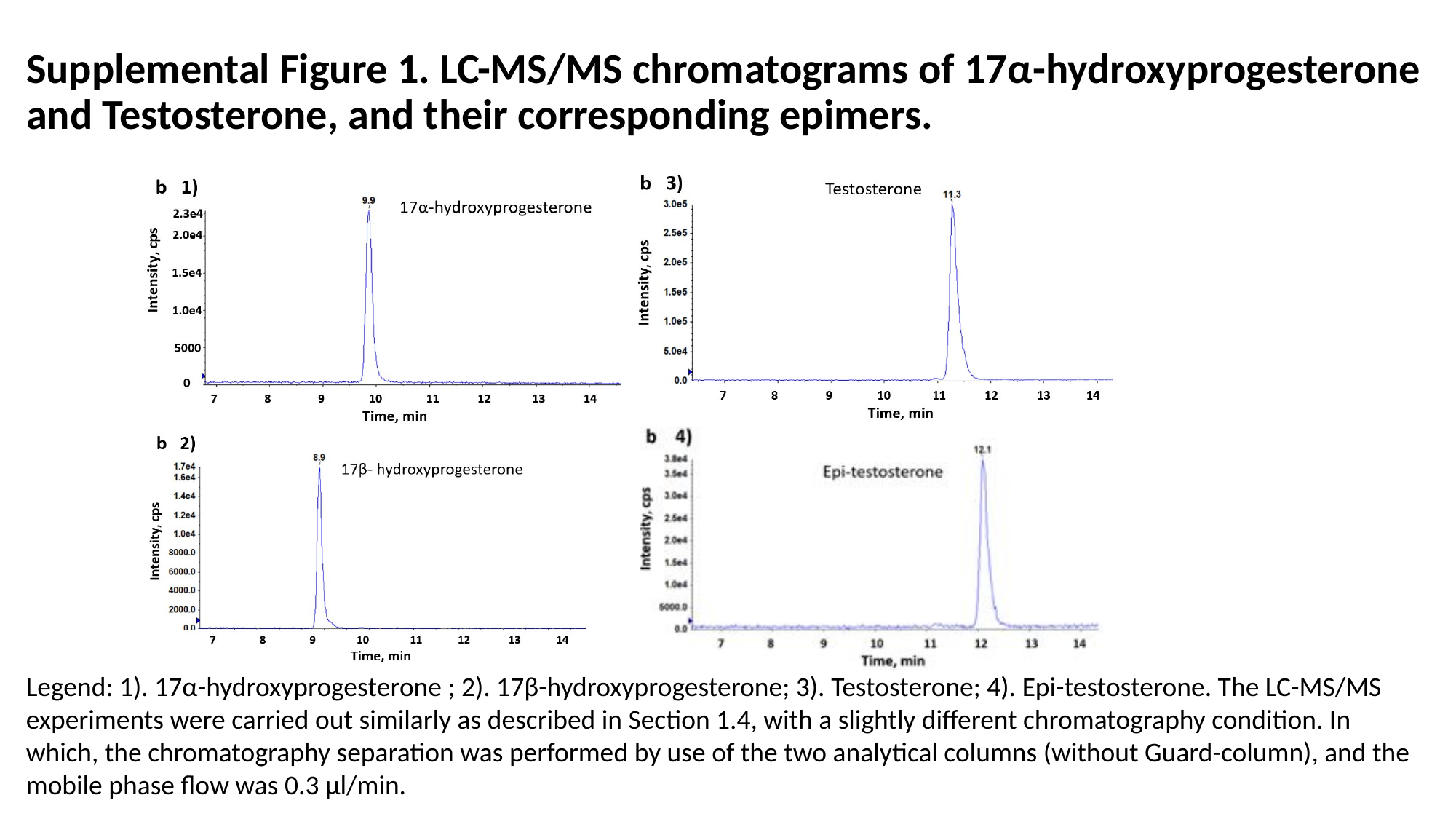

# Supplemental Figure 1. LC-MS/MS chromatograms of 17α-hydroxyprogesterone and Testosterone, and their corresponding epimers.
Legend: 1). 17α-hydroxyprogesterone ; 2). 17β-hydroxyprogesterone; 3). Testosterone; 4). Epi-testosterone. The LC-MS/MS experiments were carried out similarly as described in Section 1.4, with a slightly different chromatography condition. In which, the chromatography separation was performed by use of the two analytical columns (without Guard-column), and the mobile phase flow was 0.3 µl/min.
